# Present and Future Perspectives on Therapeutic Options for Carbapenemase-Producing *Enterobacterales* Infections

**DOI:** 10.3390/microorganisms9040730

**Published:** 2021-03-31

**Authors:** Corneliu Ovidiu Vrancianu, Elena Georgiana Dobre, Irina Gheorghe, Ilda Barbu, Roxana Elena Cristian, Mariana Carmen Chifiriuc

**Affiliations:** 1 Microbiology Immunology Department, Faculty of Biology, University of Bucharest, 050095 Bucharest, Romania; ovidiu.vrancianu@yahoo.com (C.O.V.); dobregeorgiana_95@yahoo.com (E.G.D.); ilda.czobor@yahoo.com (I.B.); carmen.chifiriuc@bio.unibuc.ro (M.C.C.); 2 The Research Institute of the University of Bucharest, 050095 Bucharest, Romania; 3 Department of Biochemistry and Molecular Biology, Faculty of Biology, University of Bucharest, 050095 Bucharest, Romania; roxana.cristian95@yahoo.com

**Keywords:** Carbapenem-resistant *Enterobacterales* (CRE), multi-drug resistance, novel antibiotics

## Abstract

Carbapenem-resistant *Enterobacterales* (CRE) are included in the list of the most threatening antibiotic resistance microorganisms, being responsible for often insurmountable therapeutic issues, especially in hospitalized patients and immunocompromised individuals and patients in intensive care units. The enzymatic resistance to carbapenems is encoded by different β-lactamases belonging to A, B or D Ambler class. Besides compromising the activity of last-resort antibiotics, CRE have spread from the clinical to the environmental sectors, in all geographic regions. The purpose of this review is to present present and future perspectives on CRE-associated infections treatment.

## 1. Introduction

**Figure 1:**
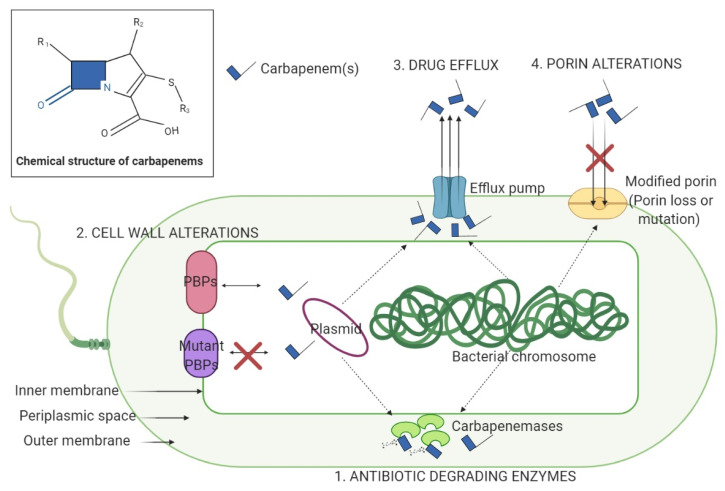
Major resistance mechanisms associated with carbapenem-resistant *Enterobacterales* (CRE) infections.

In 2017, the World Health Organization (WHO) published a list of resistant bacteria against which there is an urgent need to develop new antibiotics [1]. Critical priority bacteria included carbapenem-resistant *Enterobacterales* (CRE). These bacteria are among the most common pathogens associated with severe infections, such as sepsis, pneumonia, urinary tract, and intra-abdominal infections, having, which along with the current COVID-19 pandemic are having a major impact, both clinically and economically [2]. Initially, *Enterobacteriaceae* posed a threat to public health because of their ability to resist the action of β-lactam antibiotics (BLAs) by producing broad-spectrum β-lactamases. At first, these enzymes inactivated penicillins, but later, due to the introduction of new antibiotics for the treatment of infections, their spectrum expanded. This situation has led to the intensive use of carbapenems as first-line drugs, leading to CRE’s emergence over time [3]. There are three main mechanisms of CRE resistance, i.e., enzyme production (extended-spectrum beta-lactamases (ESBLs), metallo-beta-lactamases (MBLs) and other categories of carbapenemases), efflux pumps, and porin mutations [4,5]. Depending on the type of resistance mechanism, CREs are divided into carbapenemase-producing CREs (CP-CREs) and non-CP-CREs. CP-CRE can produce a large variety of carbapenemases which can be divided into three groups according to the Ambler classification: class A, class B and class D ß-lactamases [4]. Regarding the fourth class, the clinical relevance of C Ambler class enzymes remains to be solved [5]. CRE can accumulate many carbapenem resistance mechanisms, such as porin loss, efflux pump overexpression and changes in penicillin-binding proteins [6,7] (Figure 1). While carbapenemases specifically target carbapenems and other categories of BLA, efflux pumps and porin changes are associated with the presence of multidrug resistance (MDR) [8], blocking the penetration of different antibiotics into the bacterial cell.

In response to the CRE threat, efforts have been made to reduce the spread and prevent the development of resistance, reducing nosocomial infections associated with CRE by up to 60% by the end of 2020 [9]. The Infectious Diseases Society of America has launched the “10 × 20” campaign to develop 10 new antibiotics by the end of 2020, with two such antibiotics (e.g., telavancin and ceftaroline fosamil) already receiving FDA approval [10]. In this review, old and current antibiotics, and future promising perspectives that are currently under investigation for winning the war against the emerging CREs are briefly discussed.

## 2. CRE Permeability and the Impact of Porin Deletions and Efflux Pumps

The efficiency of antibacterial agents is related to their capacity to reach inhibitory concentrations in the vicinity of their target, an aspect that is particularly challenging in Gram-negative bacteria, due to their complex envelope structure [11]. The Gram-negative envelope consists of an inner membrane (IM), which is a symmetric phospholipid bilayer, a thin peptidoglycan (PG) layer, and an outer membrane (OM). The OM acts as a barrier to hydrophobic and hydrophilic compounds, including nutrients, metabolic substrates, and antimicrobials, but access is provided by the porins and water-filled channels [12,13]. For example, in *Escherichia coli*, OmpF and OmpC porins are size restricted and allow the access of hydrophilic charged compounds, including β-lactams and fluoroquinolone antibiotics. RND (Resistance-Nodulation-cell Division) efflux pumps, such as AcrAB-TolC in *E. coli*, have a significant role in eliminating antibiotics from the periplasm [12].

OMP proteins are generally classified into high molecular weight OmpC and low molecular weight OmpF. Deletion of OMP proteins could cause bacterial resistance. In *Enterobacterales*, resistance to carbapenems may be associated with the deleting of OMP proteins, both in the presence and absence of β-lactamase production. The antibiotic enters the bacterial cells through OMPs, which function as porous channels. When deletion or reduction of these proteins occurs, the permeability of the OM decreases, leading to increased bacterial resistance by blocking access to the cell. *Enterobacterales* such as *Enterobacter cloacae* and *E. aerogenes* show OmpK35 and OmpK36 analogs with OmpC and OmpF, as well as OmpD [14]. OMPs are either poorly expressed or their structure is modified, being replaced with other types of protein. In the study by Lee et al., 357 *Enterobacter* isolates were analyzed. Of these, 31 isolates were resistant to imipenem. In the case of imipenem-resistant *E. cloacae* and *E. aerogenes* isolates, the expression of OmpD and OmpK35 proteins was also decreased. Thus, it was observed that the primary mechanism of resistance to imipenem was decreased expression of OMPs [14]. In the study by Ye et al., 78 CRE strains were analyzed for carbapenem resistance mechanisms. The authors detected OMPs in CRE strains, demonstrating the relationship between carbapenem resistance and OMP protein deletion [15]. In an observational study of patients with CRE infections treated with meropenem-vaborbactam (MER-VAB), the impact of some porin mutations was evaluated. In the case of isolates harboring porin mutations, the required MICs were higher. The two most common mutations identified in this study among KPC-producing *Klebsiella pneumoniae* clinical isolates have been shown to have varying effects on the permeability of the OM. Mutant *ompK36* with a glycine–aspartic acid insertion at position 134 leads to a constricted inner porin channel, whereas an IS*5* promoter insertion results in decreased *ompK36* expression [16,17]. Consequently, the presence of these mutations may be associated with increased bacterial resistance to MER-VAB therapy. Miao et al. evaluated 67 CRE isolates consisting of *K. pneumoniae*, *K. oxytoca*, *K. aerogenes*, *Serratia marcescens*, *E. cloacae*, *Raoultella ornithinolytica*, and *E. coli*. Regarding the presence of porin mutations, mutations in OmpK35 and OmpK36 porins were detected in *K. pneumoniae*. In *E. cloacae*, *S. marcescens*, and *E. coli*, mutations in OmpF porin were detected. Therefore, resistance to carbapenems can be mediated by the cumulation of enzymatic and non-enzymatic mechanisms in the same strain [18].

## 3. Old Antibiotics in Treating CRE Infections

Although most antibiotics have limitations in the treatment of CRE infections, a number of old antibiotics reintroduced into the therapeutic arsenal in recent years are still active. 

For challenging Gram-negative resistant bacteria, polymyxins and tigecycline were considered an option, but resistance to these antibiotics is increasing alarmingly [19,20,21,22,23,24,25]. 

Additionally, phosphomycin and aminoglycosides are used occasionally [26]. Carbapenems still play an important role in treating CRE, either in high doses in combination with other active agents, or in dual therapy. Carbapenems remain some of the most potent classes of antibiotics employed in the last line of defense against nosocomial MDR infections. Carbapenems are BLAs, which differ from penicillin by substituting a carbon atom with a sulfur atom at the C-1 of the five-membered penicillin-like ring and adding a double bond to the β-lactam nucleus of penicillins. However, the emergence and diversification of β-lactamases remains a major obstacle to the effectiveness of carbapenems. Old antibiotics such as minocycline, doxycycline, sulfamethoxazole (SXT), and chloramphenicol may be effective against certain CRE isolates [27,28]. 

A known example is colistin, administered systemically as colistin methane-sulfonate (CMS). Colistin is an important cationic antimicrobial peptide, reintroduced for clinical treatments in the 20th century due to the increase in bacterial resistance and the lack of new effective agents [29]. In the old literature, it was reported that colistin was associated with high rates of toxicity, such as neurotoxicity, nephrotoxicity, and neuromuscular blockade, sometimes leading to fatal consequences [30]. Polymyxins have been associated with several neurotoxic adverse effects, including facial and peripheral paresthesia, dizziness, muscle weakness, confusion, vertigo, and neuromuscular blockade, leading to respiratory failure, convulsions, apnea, and coma [31]. Death was attributed to colistin therapy in less than 5% of cases [32]. Consequently, colistin use was limited in the late 1970s, except for the treatment of patients suffering from cystic fibrosis (CF) and in topical solutions with other antimicrobial agents to treat ear or eye infections [33]. The spectrum of action of colistin includes Gram-negative MDR and extensively-drug resistant (XDR), especially *K. pneumoniae*, *Acinetobacter baumannii* and *Pseudomonas aeruginosa*. In the International Network for Optimal Resistance Monitoring (INFORM) global surveillance program from 2012 to 2015, a total of 24,750 *Enterobacteriaceae* isolates was investigated, including 24,619 MBL-negative and 131 MBL-positive. It was observed that 99.4% of the *Enterobacteriaceae* isolates showed susceptibility to the new combination ceftazidime-avibactam (CAZ-AVI), and 82.8% showed susceptibility to colistin [34]. In recent years, the complex characterization of colistin’s pharmacokinetic properties demonstrated its superior activity compared with CMS, thus positioning colistin in a revolutionary period [35]. Currently, the clinical efficacy of colistin can be observed in several clinical trials focused on bacteremia or ventilatory-associated pneumonia (VAP) produced by MDR or XDR pathogens. The main limitations of the published studies are the non-randomized design with a variety of dosages, their retrospective nature, and the absence of a loading dose, whereas the simultaneous administration of other active in vitro antibiotics renders inconclusive the efficacy of monotherapy with colistin [35]. In CRE infections, the combination of colistin with another active antibiotic has been reported to be beneficial in terms of survival, as depicted in the systematic review conducted by Karaiskos et al., which included 19 studies and a total number of 3201 CPE patients [36]. The combination of meropenem with gentamicin or tigecycline or colistin led to a significant minimization of mortality especially in patients with high mortality levels and septic shock [37]. However, many clinicians, taking into consideration the pharmacokinetic/pharmacodynamics limitations of colistin and the development of resistances, favor combination treatment [38]. 

Colistin resistance may be due to the presence or acquisition of mobile genetic elements, especially plasmids harboring *mcr* genes. In 2015, the first plasmid-mediated colistin resistance was detected in an *E. coli* strain collected from food animals [39]. However, colistin resistance mediated by plasmids has been reported worldwide [40]. The *mcr-1* gene acts through modifying the OM lipopolysaccharides (LPS) by an phospho-ethanolamine transferase (pEtN transferase), which mediates the addition of pEtN to lipid A [41]. Generally, *E. coli* strains harboring the *mcr-1* gene exhibit a low-level of colistin resistance [42]. Another gene involved in colistin resistance is mcr-2 gene, identified by Xavier et al. in *E. coli* strains in Belgium. The mcr-1 and mcr-2 proteins showed 80.65% identity [43]. In 2017, Yin et al. described a third mobile colistin resistance gene, *mcr-3* [44]. In 2017, Carrattoli et al. detected a new plasmid-mediated colistin gene, *mcr-4*, in *Salmonella* on a small, non-self-conjugative plasmid [45]. In 2017, Borowiak et al. described a novel transposon-associated pEtN gene, *mcr-5*, which conferred colistin resistance in d-tartrate-fermenting *S. enterica* subsp. *enterica* serovar Paratyphi B [46]. Recently, Carroll et al. have described the *mcr-9* gene, a novel *mcr* homolog detected in MDR colistin-susceptible *S. enterica* serovar Typhimurium strain [47]. Insertion sequences (IS), the smallest (0.7–2.5 Kb) and simplest transposable elements found in bacteria, are also involved in colistin, as well as carbapenem, resistance [48]. The most common mechanism for developing colistin resistance is inactivation of the *mgrB* gene in *K. pneumoniae*, following the transposition of different types of IS, such as IS903, ISKpn26, IS10R, and IS5 [49]. In colistin-resistant strains of *Klebsiella* sp. and *E. coli*, alteration of *phoP* (a member of the two-component system phoP/phoQ involved in bacterial virulence, by mediating the Mg2+ transport and other physiological processes in several Gram-negative species) and *mgrB* (the regulator of the phoP/phoQ system) genes and by different IS, such as IS1, ISKpn14, ISKpn28, IS903, IS5, IS3, and ISEc69, can sometimes induce a pan-drug-resistance (PDR) phenotype [50,51,52].

Fosfomycin, discovered in 1960, was used for a short time and then discontinued due to the appearance of other agents and the development of antimicrobial susceptibility tests. Since then, Fosfomycin has been used in numerous countries for various indications, both in its intravenous (disodium salt) and oral formulations (calcium salt or trometamol). In recent years, the use of Fosfomycin has increased due to the notable incidence of MDR microorganisms for which Fosfomycin constitutes, alone or in combination, a treatment alternative [53,54]. Fosfomycin disodium is mainly used to treat UTIs, particularly those caused by *E. coli* and *Enterococcus faecalis*, and, in combination with other antibiotics in the treatment of nosocomial infections caused by resistant Gram-positive and Gram-negative bacteria [55]. In the case of oral Fosfomycin, in most European countries it has been used mainly in treating uncomplicated cystitis or other UTIs, particularly those caused by *E. coli* and *E. faecalis*. In the USA, the Food and Drug Administration (FDA) has approved oral Fosfomycin only for uncomplicated UTIs. Oral Fosfomycin is approved in several European countries for the therapy of soft-tissue infections and sepsis [56]. However, it should be noted that the intravenous formulation of Fosfomycin is available in five countries in Europe, i.e., Spain, France, Germany, Austria, and Greece [57]. 

The lack of a consensus regarding the determination of Fosfomycin breakpoints between the Clinical and Laboratory Standards Institute (CLSI) (≤64 mg/L) [58] and the European Committee on Antimicrobial Susceptibility Testing (EUCAST) (≤32 mg/L), the higher MIC required by several microorganisms (*Klebsiella* spp., *Enterobacter* spp., *Serratia* spp., *P. aeruginosa*) and the differences between the appropriate concentrations needed against Gram-positive and Gram-negative bacteria have resulted in recommended dosages for treating MDR microorganisms infections that vary between 8 and 12 g/day for Gram-positive bacteria and 16 and 24 g/day for Gram-negative bacteria [59,60]. According to CLSI, the only approved MIC method for testing Fosfomycin susceptibility is agar dilution, using Mueller–Hinton agar plates supplemented with 25 mg/L of glucose-6-phosphate, whereas broth microdilution MIC testing should not be performed [58]. However, because agar dilution is a laborious and time-consuming method compared to other susceptibility tests, it is not routinely used in microbiology laboratories. Although broth microdilution is not an inadvisable technique for Fosfomycin, it is the base of most automated susceptibility tests. Moreover, different studies have shown a lack of correlation in Fosfomycin susceptibility among broth microdilution, E-test, or disc diffusion tests [61,62,63]. Ballestero-Téllez et al. investigated the influence of the inoculum size used in agar dilution and broth microdilution, according to CLSI, in Fosfomycin MIC discrepancies. It was observed that susceptibility testing, either in broth or agar, should be done using higher bacterial inocula to capture the bacterial population’s whole resistance [64]. More recent studies demonstrate that different susceptibility testing tools can give very different results regarding Fosfomycin resistance, with false positives being an alarming problem that may unnecessarily limit the use of this agent [65,66,67,68,69]. However, these studies show that agar dilution remains the gold standard in determining the MIC values of Fosfomycin.

Phosphomycin has bactericidal activity against susceptible microorganisms and is a unique mechanism for inhibiting the first phase of peptidoglycan synthesis [70]. Phosphomycin disodium has several toxic effects, such as hypocalcemia and increased sodium levels, but some effects can be controlled in the clinic. In in vitro studies, phosphomycin was active against 80% of *Staphylococcus aureus*, *E. faecium*, *E. coli*, and *K. pneumoniae*, and less active against CR strains of *K. pneumoniae* [71]. In the case of 396 strains of *K. pneumoniae*, isolated from hospitals in Greece, phosphomycin was the third most effective agent [72], results confirmed in a study of strains from hospitals in the USA [73]. Of great interest is the lack of efficacy of phosphosmycin against *A. baumannii* [74]. The in vitro development of phosphomycin resistance during treatment is a problem in the clinic, so it is recommended to use it in combination therapy [73,74]. These studies demonstrate that there is still room for the use of phosphomycin in the clinic against CRE infections [36,75]. However, the biggest challenge related to phosphomycin is the need to target CRE, XDR, and PDR strains. In the case of CRE infections, the use of combination therapy is a basic rule. In the case of phosphomycin, use in combination therapy is recommended to block the selection of resistant bacteria. The recent study called ZEUS [76] led to a change in this dogma, as it was observed that there are groups of patients who respond differently to combination therapy. Regarding the choice of agent for use in combination therapy with phosphomycin, it has been observed that aminoglycosides may increase the efficacy of therapy [77]. However, in a randomized clinical study, the addition of nebulized amikacin-phosphomycin to the treatment of VAP did not improve its efficacy [78]. No synergistic action has been observed between phosphomycin and colistin, but there are studies that have shown synergy with other carbapenems [79].

Tigecycline has been used in recent years to treat infections caused by CRE and carbapenem-resistant-*A. baumannii* [80]. Low serum concentration and low penetration of the agent into the epithelial lining fluid have decreased the efficacy of tigecycline [81]. In the case of VAP, hospital-acquired pneumonia (HAP), and infections caused by *A. baumannii*, it was recommended to use a higher dose of tigecycline. However, all these situations are outside the routine use of tigecycline [82]. Elevated tigecycline concentrations have been observed to be associated with decreased fibrinogen levels and gastrointestinal side effects [83,84]. Due to the characteristics of this drug and the high mortality rate when administered as monotherapy to severely ill patients, the use of tigecycline in combination therapy is recommended. Before the advent of state-of-the-art antimicrobial agents, the agents used in combination therapy were colistin and meropenem [80,85]. In the era of the new antibiotics, tigecycline will remain a valuable antibiotic, especially in combination therapy. However, further studies are required in order to define new possible combinations.

The emergence of CRE isolates has brought aminoglycosides to the frontline since an aminoglycoside seems the appropriate antimicrobial to which CRE isolates show in vitro susceptibility [86]. The susceptibility of bacterial strains to the action of aminoglycosides varies depending on the presence of aminoglycoside-modifying enzymes (AMEs) that have different affinities for different aminoglycosides [87,88,89,90]. Genes encoding AMEs can be transferred as part of gene cassettes in the case of integrons, as well as through conjugation mechanisms [91]. Furthermore, methylation of 16S rRNA by *armA*, the most prevalent methylase [92], confers a high level of resistance to almost all aminoglycosides [93]. Aminoglycosides are among the most important antibiotic classes used to treat nosocomial infections caused by *A. baumannii* strains [94]. In general, aminoglycosides are used in combination therapy and as monotherapy in urinary tract infections. In the case of urinary tract infections, the response rate was 88% when given alone and 64% when given in combination with other polymyxins [95]. The use of aminoglycoside therapy for infections located in other parts of the body is difficult due to the pharmacological and pharmacokinetic properties of aminoglycosides. The use of aminoglycosides alone for the treatment of CRE infections has been associated with extremely high mortality rates of approximately 80% [8]. There are also a number of other disadvantages that prevent the optimal use of aminoglycosides. In the case of many MDR and XDR pathogens, borderline susceptibilities against these breakpoints have been observed, a fact further complicated by the difficulty in predicting pharmacokinetic/pharmacodynamics properties of aminoglycosides in critically ill patients [86,96]. According to the pharmacokinetic/pharmacodynamics data, monotherapy with aminoglycosides could be very risky for critically ill patients especially in infections in compartments outside the urinary tract [8,97]. Short duration courses of aminoglycosides (5–7 days) given as once-daily regimens are associated with less nephrotoxicity compared to multiple daily dosing [98]. When combination treatment is considered, aminoglycosides have been combined with almost all classes of antibiotics [86]. However, more clinical research is needed on these observations.

## 4. Current Promising Antibiotics in Treating CRE Infections

In response to increasing resistance, several new antibiotics have been developed that target KPC (*K. pneumoniae* carbapenemase)—producing *Enterobacteriaceae* and other CRE isolates. Other antibiotics in the final stages of development target pathogens that produce metallo-β-lactamases (MBL) and MDR *A. baumannii* [29]. Currently, CAZ-AVI, ceftolozane/tazobactam (CEF-TAZ), MER-VAB, and eravacycline have been included in clinical therapy in America and Europe, while plazomicin has received FDA approval [29]. 

Selecting an antimicrobial compound for CR Gram-negative infections is almost always challenging, although the degree of difficulty varies depending on the specific clinical scenario. The major issues associated with carbapenemase production are clinical, due to compromising the last-resort antibiotics activity used for treating serious infections, and epidemiological, due to their dissemination into various bacteria across almost all geographic regions and their spread from clinical to environmental reservoirs. An important advancement is the newly launched antibiotics targeting some of the current most problematic Gram-negative pathogens, including CRE. CAZ-AVI and MER-VAB have become important contemporary treatment options for CRE infections in countries where these agents have become available for clinical use [99].

CAZ/AVI, marketed as Allergan, is an intravenous combination of a third-generation cephalosporin and a 2015 FDA-approved β-lactamase inhibitor. CAZ/AVI is used for the treatment of a wide range of bacterial infections, including cUTI, complicated intra-abdominally infections (cIAI), BSI, and pneumonia, as well as hospital-associated and ventilator-associated pneumonia (HAP/VAP) [100,101,102]. The effectiveness of this combination is based on the addition of avibactam, which is a non-β-lactam β-lactamase inhibitor that reinforces the activity of ceftazidime against class A (KPCs) and C (AmpC) β-lactamases, as well as certain class D enzymes (OXA48) [29]. It is also worth mentioning that avibactam has the advantage of being recycled during successive acylation-deacylation cycles undergone by β-lactamases [103]. However, CAZ-AVI’s effectiveness is often limited by the occurrence of MBLs and mostly class D enzymes [101,104].

The efficacy and tolerance profiles of CAZ/AVI have been investigated in many clinical trials and experiments. A study conducted by the International Network for Optimal Resistance Monitoring (INFORM), which integrated more than 30,000 strains of *Enterobacteriaceae* isolated from patients with various bacterial infections, revealed an increased susceptibility of these microorganisms to CAZ/AVI combination (99.5%). According to these observations, the MIC90 for CAZ/AVI was estimated to be about 0.5 μg/mL, lower than the MIC90 required for cephalosporins to inhibit the growth of 90% of bacterial species. Notably, of the 185 cases resistant to CAZ/AVI, one-third were MBL-producers, emphasizing the clinical implications of this molecular mechanism and the need for vigilance in clinical practice [104]. Another study demonstrated the superior efficacy of CAZ/AVI compared to different therapeutic regimens incorporating carbapenem and an aminoglycoside (CB + AG) (*n* = 25) and a carbapenem with colistin (CB + COL) (*n* = 30) in managing CR *K. pneumoniae* strains [105]. It has also been observed that CAZ/AVI can improve 90-day survival rates to 90% versus 56% and 63%, respectively, for patients treated with CB + AG and CB + COL [105]. CAZ/AVI showed an excellent safety profile. The nephrotoxicity of CAZ/AVI was much lower (18%) compared to other combinations, making CAZ/AVI a promising treatment for the therapeutic management of CR *K. pneumoniae* strains. Notably, another study, led by van Duin et al., 2018, showed that CAZ/AVI was associated with a slight improvement in mortality rate in CRE patients, from 32% associated with colistin use to 9% [106].

Several recent studies have also revealed certain molecular mechanisms that may be associated with CAZ/AVI resistance. Some of these studies have shown that mutations in the Ω-loop of the KPC enzyme may lead to higher amounts of ceftazidime hydrolysis that cannot be overcome by avibactam. Other studies have suggested the existence of alternative mechanisms involving either the production of MBLs [104], mutations in different KPC enzymes [107,108], decreased outer membrane permeability, or enhanced drug efflux [109]. However, a relatively recent study led by Huang et al. demonstrated that the optimal therapeutic approach for CAZ-AVI-resistant isolates depend on the antibiotic resistance mechanism triggered. Notably, it has been shown that the combination of CAZ/AVI and meropenem is active and may suppress the recurrence of CAZ/AVI-resistant, meropenem-susceptible KPC-producing *K pneumoniae* isolates. Besides, they reported that clinically achievable CAZ/AVI concentrations might be satisfactory in suppressing the regrowth of CAZ/AVI resistant KPC-producing *K. pneumoniae* with Omp mutations and higher bla_KPC_ levels [110].

CEF-TAZ is a new semisynthetic antipseudomonal cephalosporin used in the treatment of cUTIs, cIAF, and HAP. CEF is an oxyimino-amino-thiazolyl cephalosporin, very similar structurally to CAZ but with a modified side chain that contributes to its stability in the presence of AmpC β- lactamases, prevents the hydrolysis of the β-lactam ring, and thus confers potent activity against *Enterobacteriaceae* strains [111]. CEF-TAZ has in vitro activity against *Streptococcus* species and has diminished activity against *S. aureus* strains. CEF-TAZ also has improved activity against a significant number of species belonging to the *Enterobacteriaceae* family such as *E. coli*, *K. pneumoniae*, *Enterobacter* spp., *Citrobacter* spp., *Serratia* spp., *K. oxytoca*, and *P. mirabilis* [112,113]. It has also been demonstrated that CEF-TAZ has in vitro activity against *Bacteroides fragilis*, *Prevotella*, and *Fusobacterium* spp. The in vitro activities of CEF-TAZ were determined for 1774 isolates of *Enterobacteriaceae* collected by 30 medical centers from the China Antimicrobial Surveillance Network (CHINET) in 2017. CEF-TAZ demonstrated potent activity against almost all *Enterobacteriaceae*, including K. pneumoniae and E. coli, with a susceptibility rate of 94.6% [111]. In the study conducted by Saran et al., CEF-TAZ activity was investigated in the case of 60 Enterobacteriaceae strains isolated from samples obtained from hematological patients in Poland. The results demonstrated that this combination could be a good option in treating CRE infections [114]. Castanheira et al. evaluated the activity of CEF-TAZ against 2362 *Enterobacteriaceae* isolates obtained from respiratory tract specimens from patients hospitalized in the United States during 2013 to 2015. CEF-TAZ inhibited 90.6% of the 2362 *Enterobacteriaceae* isolates, including *K. pneumoniae, K. oxytoca, E. aerogenes, E. cloacae, E. coli* and *Proteus mirabilis* [115]. In another study, 733 ESBL *Enterobacteriaceae* isolates including 486 *E. coli*, 190 *K. pneumoniae* and 42 *E. cloacae* were investigated regarding susceptibility to CEF-TAZ. CEF-TAZ inhibited 95.5% of the *E. coli* isolates but only 83.0%, 64.3%, and 80.0% of *K. pneumoniae* and *E. cloacae* [116].

Relebactam and varbobactam, some of the best-studied BLIs, showed strong in vitro efficiency over class A and C β-lactamases [117]. Recently, they have become increasingly used in the therapeutic management of CRE in combination with carbapenems, such as imipenem (with relebactam: IMI/REL) and meropenem (with varbobactam: MER/VAB) [118,119]. IMI/REL gained FDA approval in July 2019, whereas MER/VAB was approved for use in the USA in 2017 [118]. Interestingly, it has been observed that adding REL, an AVI-like BLI, to IMI can increase the susceptibility to IMI of CRE isolates from 8% to 88% [120]. Similar observations were made on strains of *P. aeruginosa* [121,122]. However, information on CRE susceptibility levels to IMI/REL is conflicting: for example, a study conducted by Livermore et al. demonstrated a modest efficacy of IMI/REL on *K. pneumoniae* VIM, IMP, and NDM-producing strains [123]; in contrast, two other independent studies reported a 100% susceptibility to *K. pneumoniae* producing KPC-2 and KPC-3 isolates [120,124]. IMI/REL also proved its potency against CAZ/AVI-resistant *K. pneumoniae* isolates that harbored OXA-48-type carbapenemase; yet, this study highlighted that IMI/REL and CAZ/AVI have overlapping spectra of action and that these observations can be harnessed to improve the therapeutic management of CRE infections [120]. In the study conducted by Yang et al., a total of 8781 *Enterobacteriaceae* isolates were collected from 22 hospitals across 7 geographic regions of China. The most frequently identified *Enterobacteriaceae* species was *E. coli* (53.3%), followed by *K. pneumoniae* (33.6%) and *E. cloacae* (6.2%). The *Enterobacteriaceae* isolates showed 95.2% overall susceptibility to IMI/REL, demonstrating the effectiveness of IMI/REL treatment [125]. In a recent study (SMART Surveillance Europe 2015–2017), a total of 11,564 *Enterobacteriaceae* isolates from 22 countries has been investigated in terms of susceptibility to IMI/REL. IMI/REL inhibited ≥99% of *E. coli, K. oxytoca, Citrobacter freundii* and *K. aerogenes* isolates, and 92.2–97.4% of *K. pneumoniae, E. cloacae* and *S. marcescens* [126]. 

Another recent addition to the therapeutic arsenal against CRE infections is the meropenem-varbobactam (M/V) combination. This combines a carbapenem with varbobactam (or RPX-7009), a pharmacophore-based boronic acid BLI active against Ambler class A and C β-lactamases. The FDA approved m/V, marked as Vabomere, in August 2017 to treat cUTIs, including pyelonephritis caused by CRE in patients 18 years of age and older [118,127]. The M/V combination’s efficacy and safety were tested in two clinical trials, TANGO I and TANGO II, showing results as good as those of other compounds and antibacterial combinations used in the management of CRE infections [128,129]. Recently, however, M/V has gained approval from the European Medicines Agency (EMA) for the treatment of cUTI, acute pyelonephritis, cIAIs, HAP, VAP, as well as other infections caused by aerobic Gram-negative pathogens in adults with limited treatment options [130].

Cefepime/zidebactam (WCK 5222) revealed in vitro antimicrobial activity against *Enterobacteriaceae*. Zidebactam is a non-β-lactam bicyclo-acyl hydrazide with two mechanisms of action, direct inhibition of β-lactamases and PBP2 inhibition [131]. The spectrum of action is broad, acting against all four β-lactamase classes (A, B, C, and D), except MBLs. Zidebactam binds to PBP2 with high affinity, while cefepime binds PBP3 and less PBP2 and PBP1a/1b. The primary mechanism of action of zidebactam/cefepime is the improvement of the antibiotic’s action by complementary binding to PBPs [132]. In the study conducted by Karlowsky et al., the in vitro susceptibility of 1018 clinical isolates of non-carbapenem-susceptible *Enterobacterales*, collected worldwide (49 countries) from 2014 to 2016, to cefepime-zidebactam has been investigated. Cefepime-zidebactam inhibited 98.5% of non-carbapenem-susceptible *Enterobacterales*. Moreover, cefepime-zidebactam was active against the majority of *Enterobacterales* (≥95%) isolates that were resistant to ceftazidime-avibactam CEF-TAZ, IMI-REL, and colistin. The results from this study support the continued development of cefepime-zidebactam as a potential therapy for infections caused by *Enterobacterales* [133]. A total of 2228 non-duplicate clinical isolates of *Enterobacterales* were collected from 45 medical centers across China in the CHINET Program in 2018. The study aimed to determine the activities of cefepime-zidebactam by broth microdilution as recommended by the Clinical and Laboratory Standards Institute. Cefepime-zidebactam demonstrated potent activity against almost all *Enterobacterales* (MIC50/90, 0.125/1 mg/L), including *bla* KPC-2-positive *Enterobacterales* and *bla* NDM-positive *Enterobacterales* [134]. The safety, efficacy, and tolerability of these agents have already been performed in phase I by intravenous administration to healthy adult patients (ClinicalTrials.gov registration no. NCT02674347 and NCT02707107). A clinical study conducted by Rodvold et al. analyzed the zidebactam/cefepime levels in alveolar macrophage, plasma, and epithelial-lining fluid. Intravenous administration of these compounds led to moderate adverse reactions. However, the administration of WCK 5222 proved safe and well-tolerated by patients. This study demonstrated the possibility of using this combination to treat nosocomial pneumonia [135]. Cefepime-zidebactam is highly active against MBL-expressing CREs [136].

Ceftaroline/Avibactam (CPA) is a promising combination that can be used to control the spread of antibiotic-resistant pathogens. This formulation combines ceftaroline, a fifth-generation broad-spectrum cephalosporin, active on Gram-positive and Gram-negative organisms, with avibactam, an antibiotic derived from diazabicyclo-octane that can reversibly inhibit several β-lactamases, including Ambler class A, class C, and certain class D enzymes [137]. The FDA has approved Ceftaroline fosamil (Teflaro), as has the European Medicines Agency (EMA), for the treatment of acute bacterial skin and skin structure infections caused by MRSA (Methicillin-Resistant *S. aureus*) and community-acquired pneumonia (CAP) caused by MSSA (Methicillin-Susceptible *S. aureus*), MRSA, and Gram-negative bacteria [138]. The prodrug, ceftaroline fosamil, is converted to plasma in the active form; hence it binds to plasma proteins and is transported to specific sites where it exerts its antimicrobial effects binding with high affinity to all 6 PBPs, especially PBP2a [138,139]. Ceftaroline also has activity on many species of *Enterobacteriaceae* but, like other cephalosporins, it is not potent on species producing ESBL and carbapenems [140]. However, the use of ceftaroline in conjunction with avibactam (NXL104) can significantly expand the action spectrum of the combination on several MDR pathogens while maintaining its anti-staphylococcal spectrum of action [140].

CPA is being evaluated to treat bacterial infections caused by Gram-negative and Gram-positive pathogens that produce β-lactamases, CRE being of particular interest. One of the most important studies to evaluate the clinical efficacy of CPA in *Enterobacteriaceae* is that led by Castanheira et al. Among others, the authors analyzed the antimicrobial capabilities of the combination in 272 β-lactamase-producing *Enterobacteriaceae* strains, which included isolates producing ESBLs (33), carbapenemases (69), AmpC enzymes (36), and combinations of two or more enzymes (99) [140]. In the strains of *Klebsiella* spp. and *E. coli*, the most commonly reported serine carbapenemases were KPC-2 (32 strains), KPC-3 (23 strains), SME-1/-2 (seven strains), OXA-48 (five strains), KPC-4 (one strain), and NMC-A (one strain). Thirty-seven strains have been identified to co-produce AmpC enzymes and carbapenemases [140]. Interestingly, CRE isolates showed higher CPA MIC values than those of ESBL- and AmpC-producing strains (MIC50, 0.5–1 μg/mL versus 0.12 μg/mL). CPA inhibited 84% of *Enterobacteriaceae* strains at MIC ≤ 1 μg/mL, with only three carbapenemase-producing strains requiring CPA MIC values higher than >4 μg/mL [140]. Species that co-produce AmpC enzymes and carbapenemases were less susceptible to antimicrobial combinations than serine CPE strains (MIC50, 1 μg/mL compared to 0.5 μg/mL). As expected, ceftaroline/avibactam showed limited activity on MBL-producing species, especially the notorious VIM and IMP variants (MIC50 > 32 μg/mL) [140]. In parallel, the same group of authors tested the effect of ceftaroline/avibactam on a collection of 493 CPE strains, including *E. coli*, *Klebsiella* spp. and *P. mirabilis* isolates [141]. Notably, the pharmacological combination was shown to be potent in inhibiting the growth of 98.2% of strains at ≤0.5 μg/mL. In contrast, all strains were inhibited at a MIC ≤ 2 μg/mL of this combination [141]. Another recent study led by Khalid et al. revealed that administration of avibactam in conjunction with ceftaroline at 4 μg/mL might expand its spectrum of activity on *K. pneumoniae* strains bearing carbapenems and multiple β-lactamases and OmpK35 and OmpK36 porin defects [142]. Therefore, all these observations potentiate that ceftaroline/avibactam may be a useful therapeutic option in the management of CPE strains, supporting the clinical development of this pharmacological combination.

Another recently approved antibiotic in the fight against CRE is cefiderocol, also known as S-649266 [143]. Cefiderocol is a parenteral cephalosporin siderophore that shares some structural similarities with cefepime and ceftazidime. However, according to the latest data, cefiderocol has been noted as one of the most potent antimicrobial compounds in the fight against fermenting and non-fermenting Gram-negative pathogens, including CRE [144]. Cefiderocol was approved in September 2019 by the FDA to treat complicated UTIs and is currently under investigation in phase III trials regarding its effectiveness against pneumonia and other infections caused by CRE [143]. The structural peculiarities of cefiderocol give it a unique mechanism of action; cefiderocol can exploit the siderophore-iron complex pathway to penetrate the OM of Gram-negative organisms as an alternative mechanism to passive diffusion through membrane porins [139]. Once in the periplasmic space, cefiderocol dissociates from the iron complex and binds preferentially to PBP3, resulting in inhibition of bacterial growth [145]. Notably, the combination of a cephalosporin with catechol moiety has been shown to generate an antibacterial compound with many improved pharmacological properties, potent over various β-lactamases, including KPC, NDM, VIM, IMP, OXA-58, OXA-48-like, and OXA- 51-like carbapenemases, as well as ESBLs such as CTX-M [143].

We will focus here on the most relevant clinical trials and trials that have evaluated the efficacy of cefiderocol in CRE isolates. A meta-analysis conducted by Wu et al. on a worldwide collection of Gram-negative isolates revealed that cefiderocol is active on Enterobacterales spp. at MICs between ≤0.002 to 128 µg/mL, while CRE isolates showed cefiderocol susceptibility at MICs ranging between 0.008–8 µg/mL [143]. Moreover, in the ARGONAUT-I study (Antibacterial Resistance Leadership Group (ARLG) Reference Group for the testing of Novel Therapeutics), cefiderocol demonstrated in vitro superior antibacterial activity cefepime, aztreonam, ceftazidime, CAZ/AVI, and ceftolozane/tazobactam in combating CRE infections [146]. In this study, the authors also observed that the activity of cefiderocol on CRE varied depending on the type of carbapenemase expressed, with MICs of 1 mg/L for strains expressing OXA-48-like genes, 2 mg/L for KPC-3, and 8 mg/L for strains producing NDM, KPC-2, and ESBLs [146]. In line with these observations, Karlowsky et al. showed that at a concentration of ≤4 mg/L, cefiderocol possesses in vitro activity against over 99% of *Enterobacteriaceae* strains resistant to CAZ/AVI ceftolozane/tazobactam, cefepime, ciprofloxacin, and colistin [147]. Recent studies on animal infection models also support the potential utility of cefiderocol in treating infections caused by CRE [148,149]. However, the most substantial evidence regarding the therapeutic utility of cefiderocol comes from clinical trials. A recently ended, randomized, open-label, multicenter, pathogen-focused, descriptive, phase 3 trial (NCT02714595) offered remarkable insights regarding the efficacy and cefiderocol compared to best available therapy for the management of life-threatening infections caused by CR-Gram-negative bacteria, including CRE [150]. Cefiderocol showed a therapeutic efficacy similar to that of the best available therapy in the analyzed patient population. Notably, mortality was higher in the group of patients receiving cefiderocol, especially in the subset of patients with *Acinetobacter* spp. Infections [150]. However, preliminary data from a randomized, double-blind trial (APEKS-NP) in patients with nosocomial pneumonia due to carbapenem-susceptible Gram-negative pathogens showed a similar rate of mortality as compared to meropenem, providing some reassurance in the overall benefit/risk assessment [151]. Collectively, these observations support the use of cefiderocol as a potential therapy in treating CR infections while emphasizing the need to use this drug in patients without other therapeutic alternatives and in the absence of poor prognosis indicators. Undoubtedly, shortly we will find more information on the efficacy and tolerability of cefiderocol in clinical trials now underway (NCT03869437).

Aztreonam/avibactam (AZT/AVI) is a unique compound with activity against CPE and MBLs. AZT is mainly resistant to the action of MBLs; however, aztreonam can be readily hydrolyzed by Ambler class A or class C β-lactamases [152]. Notably, avibactam can inhibit these secondary β-lactamases, which is why the AZT-AVI combination may be a valuable therapeutic strategy against MβL-producing organisms with secondary β-lactamases. The combination of AZT-AVI is currently being studied in clinical trials to investigate its efficacy on MβL-related CRE infections [153]. However, both components of the combination are FDA-approved: aztreonam and CAZ/AVI, respectively. Only by using these approved drugs can patients benefit from the AZT/AVI combination; this procedure was also recently included in the Sanford guide for antimicrobial therapy of CRE infections [153]. Although the use of AZT in conjunction with CAZ-AVI is permitted for clinical use, there are still many details to elucidate regarding in vitro antimicrobial susceptibility testing (AST) of this combination or AZT-AVI [153,154].

Several studies have shown the superior efficacy of aztreonam/avibactam in CRE isolates compared to other combinations available to manage these infections. One of these studies, conducted by Chew et al., showed that the aztreonam/avibactam combination could restore susceptibility to aztreonam in a CPE collection of 13 *E. coli*, 44 *K. pneumoniae*, seven *Citrobacter freundii*, and six *E. cloacae* isolates [152]. In the first instance, 23 CPEs carrying bla_KPC2_ and bla_NDM-1_ or bla_NDM-1_, bla_NDM-5_, and bla_OXA-48_ gene variants were tested against combinations of aztreonam and meropenem with avibactam [152]. All the strains were non-susceptible to meropenem and aztreonam; however, the addition of avibactam restored the susceptibility of 95.7% of the species to aztreonam and 4% to meropenem. The other 48 CPEs were further tested against aztreonam and avibactam, administered alone or combined [152]. All isolates were non-susceptible to aztreonam; notably, the addition of avibactam restored their sensitivity to aztreonam. Overall, for the entire collection of 70 isolates, the combined MIC90s were >64 mg/L for aztreonam and 2 mg/L for aztreonam-avibactam combinations, this dose of aztreonam-avibactam formulation blocking bacterial growth for approximately 98.6% of CRE strains [152]. Another study, led by Kim et al., analyzed the in vitro activity of CAZ-AVI and AZT/AVI, respectively, while evaluating the inoculum effect on CRE strains containing both CP-CRE and non-CP-CRE strains [155]. CASE/AVI and AZT/AVI MICs were evaluated by broth microdilution using standard (1 × 10^5^ CFU/mL) and high inocula (1 × 10^7^ CFU/mL) in a batch of 81 CR *E. coli* and *K. pneumoniae* strains. The inoculum effect was defined as an ≥8-fold MIC increase with high inoculum. As many as 43% of the strains analyzed were CP-CRE, of which 34% had MBL. Notably, CAZ/AVI was active on 73% of the CRE isolates, while AZT/AVI had a lower MIC (≤8 µg/mL) against 95% of the CRE isolates. However, compared to CAZ/AVI, AZT/AVI was much less potent against non-CP-CRE isolates. The inoculum effect was much more prominent with AZT/AVI than in CAZ/AVI (47% and 18%, respectively), especially in *K. pneumoniae* isolates. However, the use of the AZT/AVI combination should be performed with caution in the clinical context. The presence of a substantial inoculum effect may contribute to the clinical failure of high-inoculum infections treated with AZT/AVI [155].

A promising therapeutic strategy in the fight against CRE, not yet validated for use in the clinical context, is to combine meropenem with nacubactam, also known as RG6080 or OP0595 [156]. Nacubactam, a bridged diazabicyclo-octanone β-lactamase inhibitor, acts on class A and class C β-lactamases while exhibiting minor activity on class D enzymes [157]. Nacubactam has an increased structural analogy with avibactam but differs from it by adding an aminoethoxy group to the carbamoyl side chain of avibactam. This change is most likely responsible for the intrinsic antimicrobial activity of nacubactam when administered as monotherapy [158]. Nacubactam is documented as inhibiting PBP2 in several *Enterobacteriaceae* strains [159]. The combination of nacubactam with other β-lactam agents acting against other PBPs can considerably increase the antimicrobial activity of nacubactam, extending its spectrum of action on *Enterobacteriaceae* producing ESBLs, AmpCs, KPCs, and MBLs, in addition to those porin-defective ESBL- and AmpC-producing *Enterobacteriaceae* [157,160].

Meropenem/nacubactam is currently being evaluated for its efficacy against CRE strains. Although the data are relatively few, the results obtained are encouraging. Barnes et al. examined the susceptibility of a collection of CR *K. pneumoniae* and *E.coli* strains harboring bla_KPC-2_ and bla_KPC-3_ β-lactamases to a panel of antibiotics, including meropenem-nacubactam at a ratio of 1:1 [158]. For the 44 CR *K. pneumoniae* strains, the MICs for these drugs varied within the collection; however, all strains were susceptible to meropenem/nacubactam. The RB1324 strain, which harbored bla_KPC-3_ genes, showed an extremely high MIC for nacubactam (>256 mg/L), most likely due to a mutation in PBP2 [158]. However, the addition of meropenem significantly reduced MIC to 0.5 mg/L, potentiating that the meropenem/nacubactam combination is superior to other BLIs in the treatment of high-resistant *K. pneumoniae* strains. In parallel, administration of meropenem/nacubactam resulted in a modest decrease in MICs for meropenem in *E. coli* strains producing the KPC variants. The most pronounced effect of adding nacubactam was observed for the strain expressing pBR322-KPC-2 wild type, which showed an approximately 30-fold reduction in MIC than MIC meropenem alone. Strains expressing P104K, V240G, V240K, and K234R variants showed a slight increase in meropenem/nacubactam MIC than strains expressing KPC-2. In contrast, foreign *E. coli* expressing the K234R variant showed no changes in meropenem MIC by referencing KPC-2 producing strain [158]. Another study, led by Monogue et al., demonstrated the therapeutic efficacy of the meropenem/nacubactam combination in *Enterobacteriaceae*-infected murine models of complicated UTIs, including NDM, KPC, OXA, CTX-M, SHV, and TEM enzyme-producing isolates. This research emphasizes that this promising combination is likely to be included soon in the antimicrobial armamentarium in the fight against CRE [156].

Plazomicin (ACHN-490) is a next-generation semisynthetic aminoglycoside derived from sisomicin [161]. Sisomicin was isolated from *Micromonospora inyoensis* in 1970 and presents a chemical structure similar to that of gentamicin. Despite the increased spectrum of activity of sisomicin compared with other clinically available aminoglycosides at the time, it was susceptible to many AMEs, limiting its use [161]. In contrast to the traditional aminoglycosides, plazomicin has several structural modifications. Plazomicin does not contain hydroxyl groups in the 3′ and 4′ positions, which protects it from two AMEs: O-nucleo-tidyl-transferase ANT(4′), which impacts both amikacin and tobramycin, and O-phosphotransferase APH(3′), which only impacts amikacin [162,163]. The introduction of the unsaturated hydroxyethyl group at position 6′ on the plazomicin molecule provides protection against N-acetyltransferase AAC(6′), which causes resistance to tobramycin gentamicin and amikacin [163]. Lastly, the plazomicin structure has an N-1 substitution with 4-amino-2-hydroxybutanoic acid, which blocks AAC(3) and ANT(2′′), which both cause gentamicin and tobramycin resistance, and APH(2′′), which causes resistance to gentamicin, tobramycin, and amikacin [164]. Plazomicin (Zemdri; Achaogen, Inc., South San Francisco, CA, USA) demonstrates in vitro activity against multidrug-resistant gram-negative pathogens and retains activity against AME-producing bacteria, in contrast to other available aminoglycosides [165,166,167,168,169,170]. Due to its enhanced spectrum of activity, the FDA designated plazomicin as a Qualified Infectious Disease Product and it was approved in 2018 to treat complicated urinary tract infections, including pyelonephritis [171]. It has been observed that plazomicin is more active than other aminoglycosides against KPC and OXA-48 producers but shows more variable activity against NDM-1-producing isolates due to the common co-production of 16S-RNA methylases in these isolates [172,173]. Cox et al. led a study regarding the activity of plazomicin on AMEs-producing *E. coli* strains. It was observed that the majority of AMEs did not impact plazomicin potency, with the exception of AAC(2′)-Ia (16-fold increase in minimum inhibitory concentration, MIC) and APH(2′′)-IVa (4- to 8-fold increase in MIC). The expression of 16S methylases also led to highly elevated plazomicin MICs (ranging from 64 to >512 μg/mL) [174]. 

The in vivo antimicrobial activity of plazomicin against *Enterobacteriaceae* has been studied in various animal models, including murine systemic [175], pulmonary [176] and UTI models [175], African green monkeys [177], and cynomolgus macaques (CM) [178].

Clinical trials completed to date include phase I, phase II and phase III clinical trials. Gall et al. conducted a phase 1, randomized, double-blind, crossover study aiming to assess the potential effects of plazomicin on cardiac repolarization (NCT01514929). Fifty-six healthy adults (24 men, 32 women) received a single therapeutic dose of plazomicin (15 mg/kg administered by 30-min intravenous infusion), a single supratherapeutic dose of plazomicin (20 mg/kg administered by 30-min intravenous infusion), placebo, or oral moxifloxacin (400 mg). Model-derived pharmacokinetic parameters and safety findings were generally consistent with other reported plazomicin studies. It was observed that therapeutic and supratherapeutic doses of plazomicin had no clinically significant effect on cardiac repolarization and were generally well tolerated [179]. Connolly et al. evaluated the efficacy and safety of plazomicin in a double-blind, comparator-controlled, phase 2 study in 145 adults with complicated UTI or acute pyelonephritis. Patients were randomized 1:1:1 to receive intravenous plazomicin (10 or 15 mg/kg of body weight) or intravenous levofloxacin (750 mg) once daily for five days. The phase 2 clinical trial results showed that the administration of plazomicin once daily for five days was an effective treatment in adult patients with cUTI, including acute pyelonephritis. Microbiological eradication was achieved in over 85% of plazomicin-treated patients in the ME population, and the investigator assessed 80% of patients who received the 15-mg/kg dose of plazomicin to be clinically cured with complete resolution of baseline signs and symptoms of infection [180]. Kuti et al. conducted a Phase 3, randomized study (CARE) evaluating the efficacy and safety of plazomicin-based combination therapy compared with colistin-based combination therapy to treat patients with bloodstream infections HAP and VAP due to CRE. These analyses included 18 patients enrolled in Cohort 1 of the CARE Study who were randomized to receive plazomicin combined with either tigecycline or meropenem, provided that plazomicin achieved its requisite pharmacodynamic exposure in all 15 patients which met the inclusion criteria [181]. Wagenlehner et al. conducted a multicenter, multinational, randomized, double-blind, phase 3 trial to demonstrate the potential activity of plazomicin and meropenem in the treatment of cUTI, including acute pyelonephritis. The results demonstrate that plazomicin was non-inferior to meropenem in treating patients with cUTIs, including acute pyelonephritis, with higher microbiologic eradication rates [182].

Eravacycline is a new, fully synthetic fluorocycline developed to treat infections caused by MDR microorganisms, such as CRE, MRSA, ESBL-producing *Enterobacteriaceae*, and VRE; in 2018, this fluorocycline was approved by the FDA and marketed as Xerava [183]. Eravacycline, formerly TP-434, was designed to overcome two of the main resistance mechanisms common to the tetracycline class: ribosomal protection, commonly seen in gram-positive organisms, and active drug efflux, common in both Gram-positive and Gram-negative organisms [184]. The safety and pharmacokinetics of eravacycline were investigated in a phase 1 clinical study [185] that included 20 patients and in a randomized phase two, double-blind, active control study, published in 2014 [186]. Two-phase 3 clinical trials (e.g., IGNITE1 and IGNITE4) recently evaluate the safety and efficacy of eravacycline [183,187].

Omadacycline (NUZYRA^®^/PTK 0796) is a new amino-methyl-cycline developed by Paratek Pharmaceuticals (Boston, MA, USA). It is an intravenous and oral antibiotic therapy, approved (2018) by the FDA, for clinical use in acute bacterial skin and skin structure infections and community-acquired bacterial pneumonia [188,189]. Recent microbiological data demonstrate omadacycline’s in vitro activity against multiple pathogens, including MSSA, MRSA, *S. pneumoniae*, β-hemolytic streptococci, VRE, *Enterobacterales*, *Legionella pneumophila*, *Mycoplasma spp., Ureaplasma* spp., *Bacteroides* spp., and *Clostridioides difficile* [190,191,192]. 

The in vivo antibacterial activity of omadacycline has been evaluated in several animal models, including neutropenic murine lung infection, thigh infection, and intraperitoneal challenge models [193,194,195].

Omadacycline has been investigated in several completed clinical trials to treat various clinical conditions. One completed phase II trial evaluated the activity of omadacycline on complicated skin and skin structure infection (cSSSI, NCT03716024) [196] and three phase III clinical trials investigated the activity of omadacycline on complicated skin and skin structure infection and community-acquired bacterial pneumonia. The phase III trials, OASIS-1 (Omadacycline for Acute Skin and Skin Structure Infections Study, NCT02378480), OASIS-2 (NCT02877927), and OPTIC (Omadacycline for Pneumonia Treatment in the Community, NCT02531438), established the non-inferiority of omadacycline to linezolid (OASIS-1, OASIS-2) and moxifloxacin (OPTIC) [197,198,199]. 

Given their broad spectrum of activity against clinically relevant Gram-positive and Gram-negative bacteria and availability as intravenous and oral formulations, fluoroquinolones are amongst the most commonly utilized classes of anti-bacterials for treating infectious diseases [200]. Delafloxacin [Quofenix™ (EU); Baxdela^®^ (USA)] is a novel, fully synthetic anionic fluoroquinolone with a modified quinolone structure that improves its spectrum of antibacterial activity, pharmacokinetic profile, and toxicity profile [201]. The chemical structure of delafloxacin is different from other fluoroquinolones based on three notable changes: a heteroaromatic ring at the N1 position that enhances its antibacterial activity, a weak polarity at the C8 position, and the absence of a primary group at the C7 position [200,202].

Delafloxacin is approved in several countries for intravenous and oral formulations to treat adults with acute bacterial skin and skin structure infections (featured indication) and/or community-acquired bacterial pneumonia [203]. Delafloxacin has demonstrated greater in vitro potency than levofloxacin against most gram-positive pathogens, including retaining activity in many levofloxacin–non-susceptible isolates. Notably, delafloxacin is 32-fold more active than levofloxacin against MRSA isolates [204] and is active both in vitro and in clinical infections against most isolates of *E. coli*, *K. pneumoniae*, *E. cloacae*, and *P. aeruginosa*, with activity similar to ciprofloxacin. 

Several phase clinical trials, including phase I, II and III trials, demonstrate that the clinical efficacy of delafloxacin is as high as that of the comparator drugs (e.g., vancomycin, moxifloxacin, ceftriaxone) in the treatment of acute bacterial skin and skin structure infections and community-acquired bacterial pneumonia, including MRSA-associated infections; furthermore, this antibiotic is as well-tolerated as the comparators [205,206,207,208,209,210,211].

A brief summary of these promising agents that include the mechanism of action, market availability, indications and limitations can be found in Table 1.

## 5. Future Promising Strategies in CRE Treatment

The scientific community has focused its efforts on identifying new strategies for combating drug resistance by repositioning non-antibiotic drugs in the antimicrobial arsenal or reconceptualizing old antibiotics.

One of the methodologies in the post-antibiotic era is the use of non-antibiotic drugs for the treatment of multidrug-resistant infections [212]. The benefits are considerable; the details of these drugs’ pharmacokinetics and toxicity are already known, and therefore the drugs can be passed directly into phase 2 of clinical trials [213]. However, the costly disadvantage of clinical trials and patent rights remains [214]. Several drugs administered either alone or in conjunction with classical antibiotics have been shown to be effective in removing resistance in CRE, such as antiretroviral compounds (Zidovudine) [215,216], antifungals (Cyclopirox) [217,218], anticancer compounds (Gallium, Mitotane, Tamoxifen) [219,220,221], and antidepressants (Sertraline) [222]. Many of these even have different therapeutic targets from conventional antibiotics, which act on DNA, the cell wall, or protein translation. Ciclopirox effectively inhibits CRE growth despite its resistant status by interfering with galactose metabolism and LPS (lipopolysaccharides) biosynthesis [217]. Gallium can inhibit ferric redox reactions and associated pathways due to its similarity to iron and can stop the bacterial growth of microorganisms resistant to the last-resort antibiotics [219].

Another promising strategy in the context of antibiotic resistance is the use of ion liquid (IL)-based antimicrobial agents. ILs are generally defined as salts composed solely of cations and anions, with melting points below 100 °C, mostly liquid at room temperature [223]. ILs are not new compounds, dating back more than a century, but have raised the awareness of the scientific community due to their tunable biological properties that allow them to be exploited to generate new and unlimited pharmacological combinations with antimicrobial effects [224]. 

Currently, ILs are categorized in different generations, with the first and second-generation IL generally focused on modulation of physicochemical properties and applications. Third-generation ILs (advanced or task-specific ILs) were based on the use of biodegradable and natural ions or ions with known pharmacological activity, such as active pharmaceutical ingredients [225]. The exploitation of ILs is currently of interest for biomedical and pharmaceutical applications. ILs antimicrobial properties fully depend on their structure, which can be adjusted by altering the cationic organic head (positive charge), the tail (usually an alkyl linear chain) and the organic/inorganic or biobased anion [226]. Antimicrobial ILs have several structural and mechanistic characteristics identical to cationic biocides, such as quaternary ammonium compounds (QACs), the primary mode of action of which is membrane-bound protein disruption [227]. 

The studies have shown that ILs target mainly the cytoplasmic (inner) bacterial membrane [228], which produce changes in the structural and dynamic properties of the outer layers [229] and subsequent disruption and loss of membrane integrity. The general mechanism of action is based on the negative charge of most bacterial cells and on the positive charge of antimicrobial ILs [230]. Consequently, in the first stage of antimicrobial action, there is an interaction between the cationic head group of ILs and the negative structural proteins of the bacterial outer layers [231]. Then, cations are absorbed into the cell layers, connecting to the cytoplasmatic membrane, and penetrating the interior of the cell [232]. ILs further lead to the coagulation of cytoplasmic constituents, causing the inhibition of crucial enzymes (i.e., AMP deaminase, acylase I, cytochrome c oxidase, glutathione reductase acetylcholinesterase, carboxylesterase, catalase), interfering with energy or self-repair processes [233], and finally causing cell death. 

Generally, ILs display antimicrobial activity toward a range of bacteria including Gram-positive cocci such as *Staphylococcus* and *Streptococcus*, Gram-positive bacteria, including *Lactobacillus* and *Bacillus subtilis*, as well as mycobacteria and fungi [229]. The most common IL cationic head groups include imidazolium, pyridinium, quinolinium, morpholinium, pyrrolidinium, and choline ions [224]. The most effective antimicrobial effects have been reported for ILs containing alkyl chains with 10–16 carbon atoms and also showing a balance between hydrophilicity and lipophilicity [234]. The use of amino acids (e.g., proline, tryptophan, phenylalanine, methionine, and valine) as anions is another approach of potential interest [224]. ILs with imidazolium cations and tryptophan anions demonstrated potent antibacterial activity against both Gram-negative and Gram-positive bacteria [235]. Ferraz et al. reported that the administration of ampicillin-based ILs inhibited the bacterial growth of several drug-resistant Gram-negative species (*E. coli*, *Klebsiella pneumoniae*), as well as Gram-positive strains of *Staphylococcus aureus* and *Enterococcus faecalis*, being superior to sodium ampicillin and bromide and chloride salts. Additionally, the administration of ILs led to the reduction in MIC (minimum inhibitory concentration) for two Gram-negative antibiotic-resistant *E. coli* strains harboring TEM and CTX M9 and CTX M2, respectively, demonstrating the potency of these ampicillin-based ILs for fighting Gram-negative resistant bugs [236]. Other studies have shown the effectiveness of combining organic cations such as choline, alkylammonium, alkyl-pyridiniums, and alkyl-imidazoliums with various inorganic anions or antibiotics (ampicillinate, carbenicillinate, oxacillinate and cephalothinate or penicillin hydrolyzate, and amoxicillin hydrolyzate) in combating the problem of antibiotic resistance in various *Enterobacteriaceae* strains [236,237,238,239]. ILs can also be combined with phage therapy or lysine therapy to strengthen the therapeutic arsenal in the context of antibiotic resistance [240]. 

Recently, Liu et al. reported syntheses of novel lignin-derived ionic liquids, with extended *N*-alkyl chains, and examined their melting points, cellulose dissolution capacities, and toxicity profiles against *Daphnia magna* and *E. coli*. New synthetic approaches to lignin-derive quaternary ammonium compounds (QAC) provided room-temperature ILs that demonstrated rapid cellulose dissolution capacity at 100 °C. The analysis of this compound confirmed that cellulose dissolution also reduced cellulose crystallinity. Broth dilution assays with ILs and *E. coli* suggested that antibacterial activity was due to both the structure of cations and type of anions. Anion toxicity followed the trend of methane-sulfonate < acetate < hydroxide. In contrast to ILs derived from syringaldehyde and 4-methoxybenzaldehyde, asymmetric methoxy substitution on the benzyl ring of the cation may have reduced the toxicity of vanillin-derived ILs [241]. The antibacterial mechanism of action for ILs is the disruption of the lipopolysaccharide cell membrane, as evidenced by the trend of increasing toxicity with increasing cation *N*-alkyl chain length. 

Important attention is also given to poly-ionic liquids (PILs), which can be designated to achieve amphiphilicity, thereby allowing the polymer’s rapid and efficient transfer through the lipid bilayer of the bacterial membrane [242]. It has been documented that PILs with high cation density and long alkyl chains have superior antimicrobial efficacy to their small-molecule counterparts [243]. However, the use of PILs is currently limited by bioaccumulation in the environment, and studies are focused on identifying compounds with optimal biodegradability [242].

Testing drugs in clinical trials to investigate their ability to kill bacteria in ways other than conventional methods is extremely laborious and expensive, and most of the time, the results are modest. Thus, the development of new methodologies that reduce costs and increase the discovery of new antibiotics is essential to reinvigorate the antibiotic pipeline. Machine learning approaches can address many of these issues associated with the synthesis, identification, and clinical validation of new compounds with antibiotic properties [244,245]. A pioneering deep-learning approach has identified new antibiotics from a pool of more than 107 million molecules, also known as the ZINC15 database. Remarkably, one of these compounds, halicin, is structurally divergent from conventional antibiotics and is potent over a wide range of microorganisms, including *Mycobacterium tuberculosis* and CRE. Halicin efficiency on pan-resistant *A. baumannii* was also confirmed by in vivo studies. This study highlighted the pivotal role of artificial intelligence approaches in describing and predicting potential candidates’ properties in reversing antibiotic resistance [246]. Additionally, some studies have highlighted the role of machine learning approaches in optimizing antibiotic combinations to reverse carbapenem resistance in Gram-negative organisms such as *A. baumannii* [247]. In a recent study, Chapman et al. combined the UV-visible spectroscopy (UV-Vis) routinely used in microbiology with principal component analysis. In this study, the full potential of UV-Vis spectrophotometry (multiwavelength collection) was used to examine bacterial growth phases when treated with antibiotics and to observe the point of resistance when an antibiotic is introduced into the media, and therefore understand the biochemical changes of the infectious pathogens. Due to the unique experimental setup and procedure that involves indirect use of antibiotics, the same test could be used for obtaining practical information on the type, resistance, and dose of antibiotic necessary to establish the optimum diagnosis and treatment for pathogenic and antibiotic resistant species [248]. In another recent study, Parvaiz et al. exploited the strategy of combination therapeutics, aiming at identifying novel β-lactamase inhibitors that can inactivate the β-lactamase enzyme of the pathogen. Inhibitor discovery applied the Site-Identification by Ligand Competitive Saturation (SILCS) technology to map the functional group requirements of the β-lactamase CMY-10 and generate pharmacophore models of active site. The authors identified certain non-β-lactam-based β-lactamase inhibitors that have the potential to be used in combination therapy with lactam-based antibiotics against MDR clinical isolates that have been found resistant against last-line antibiotics [249].

Another strategy exploited to reverse antibiotic resistance comes from synthetic biology, and involves redesigning existing antibiotics to overcome natural resistance mechanisms [250,251]. The concept is based on developing a “LEGO” set of molecular pieces that can be altered and joined together to generate larger molecules with improved antibiotic capabilities. This has been demonstrated with a new class of drugs called streptogramins, which block bacterial growth by interfering with protein synthesis. In this regard, Li et al. built new streptogramins from scratch, creating a series of modules that can be modified as needed to generate a series of variations in the structure of streptogramin molecules. After modifying and assembling these molecular LEGO pieces, it was observed that these variations had antimicrobial activity against a wide range of pathogens, including streptogramin-resistant *S. aureus*. Therefore, the synthesis and assembly of slightly modified modules can revitalize several antibiotics classes that have been abandoned due to the natural mechanisms of bacterial resistance, offering new hopes in the war against CRE [252]. Another LEGO-like approach is expected to revitalize endolysins, enzymes employed by bacteriophages to produce cell lysis and virion release, and currently used in the therapeutic management of Gram-negative bacteria [253].

The emergence and spread of *Enterobacteriaceae*, including CPE, is a significant concern. Therefore, infection control and prevention strategies are crucial for blocking the spread of MDR bacteria. These bacteria possess enzymes that hydrolyze most β-lactam and other antibiotics, leading to potentially untreatable infections [254]. Generally, transmission occurs rapidly through human and animal populations and hospitals through delayed recognition and breakdowns in infection control. Dissemination is increased by the evolution of ‘high-risk clones’, characterized by high rates of colonization, invasive disease, and transmissibility in healthcare settings. Person-to-person transmission through healthcare workers is considered the main route of transmission; however, the role of the environment in multidrug-resistant bacteria transmission is increasingly recognized [255]. CPE and those harboring ESBL can survive in biofilms within hospital drains, toilets, equipment, and other environmental niches that are difficult to access and clean. Consequently, engineering solutions, including toilet, sink, or drain removal, are necessary [256]. When CPE is recognized, environmental control should be intensified, patient surroundings should be disinfected and cleaned daily, specialized equipment should be used, and unnecessary transfers avoided. 

The correlation between hand hygiene and the prevention of the spread of disease was established in 1847 when Ignaz Semmelweis documented evidence to demonstrate the effect of hand hygiene in preventing nosocomial infections [257]. The studies show that various hand hygiene methods reduce bacterial load significantly and prevent cross-transmission and health care associated infections [258,259]. The role of hand hygiene is to prevent colonization and infection in the patient and environmental contamination. The need for hand hygiene arises whenever a microorganism transfers from one skin or inanimate surface to another surface [258]. First Global Patient Safety Challenge “Clean Care is Safer Care” (CCiSC) recommends a “five moments” approach, considered critical for meeting the needs for training, compliance measurements, and comparable performance reporting [260]. This “five moments” concept consists of a complex sequence of care which can be summarized as follows: (1) Before touching a patient; (2) Before a clean/aseptic procedure; (3) After exposure to a body fluid; (4) After touching a patient; and (5) After touching patient surroundings. The detailed recommendations can be found in the WHO Guidelines on Hand Hygiene in Health Care and the original publication [260].

In infection control and prevention strategies, antimicrobial stewardship (AMS) has a critical role. AMS deals with optimizing antimicrobial use, including dose, route, and duration, and the reduction of unnecessary use [261]. Given that up to 50% of antibiotic usage is either unnecessary or inappropriate [262], the proper administration of antibiotics is a critical factor in tackling antibiotic resistance. Prescribing the correct antibiotic at the right time and for the proper diagnosis is essential for the success of AMS strategies [261].

## 6. Discussion

According to the Global Priority List published by the WHO, CRE poses an exponentially growing threat to global public health. These pathogens have diverse and versatile mechanisms of antibiotic resistance, which complicate the clinical management of associated infections; however, the presence and diversification of β-lactamases remain the most problematic mechanism [263]. Briefly, there are at least three main groups of enzymes that mediate carbapenem resistance in *Enterobacteriaceae*, as follows: KPC (Ambler class A), MBLs (Ambler class B), and OXA-48-like (Ambler class D) β-lactamases [264]. In the USA, studies reported an increased prevalence of existing KPC and OXA-48-like producing *Enterobacteriaceae* strains; in contrast, Ambler class B enzymes are endemic in Europe [265]. Therefore, a concentration of the medical and scientific community’s efforts is needed to control the infections as effectively as possible and prevent the acquisition of resistance. Considering that several resistance mechanisms may coexist in *Enterobacteriaceae* strains, more specific approaches to these problematic infections are necessary. These strategies may involve either repurposing certain compounds and antibiotics, dual therapies with antibiotics, or reinvigorating the antibiotic pipeline with novel BLIs and antibiotics [263]. The most effective therapeutic strategy for the treatment of infections caused by CRE is still controversial. However, the scientific community’s recommendations suggest that the ideal therapy for resistant Gram-negative pathogens, including CRE, should be delivered, taking into account the mechanisms of resistance, β-lactamases present, susceptibility profiles of resistant organisms, and the severity of the patient’s illness [143,265].

Particular attention is currently being paid to new antibiotics such as CAZ/AVI, AZT/AVI, MER/VAB, plazomicin, and cefiderocol. Based on clinical data available to date, CAZ/AVI, MER/VAB, and IMI/REL are the preferred therapies for KPC-producing *Enterobacterales* due to their remarkable stability [265]. CASE/AVI is one of the most widely used therapies in the clinical management of CRE. CAZ/AVI received FDA approval in 2015 and is recommended for the treatment of a wide range of Gram-negative infections, including cUTI, complicated intra-abdominally infections (cIAI), BSI, pneumonia, as well as hospital-associated and ventilator-associated pneumonia (PAH/VAP) [100,101,102]. The effectiveness of this combination is based on the addition of AVI, which is a non-β-lactam-β-lactamase inhibitor that reinforces the activity of ceftazidime on class A (KPCs) and C (AmpC) β-lactamases, as well as certain class D enzymes (OXA48) [29]. In surveillance studies, CAZ/AVI is superior to traditional antibiotics used (e.g., carbapenem and an aminoglycoside; carbapenem plus colistin) in the management of CR *K. pneumoniae* isolates [105]. This combination showed an excellent safety profile and was associated with a slight improvement in the mortality rate in CRE patients from 32% associated with colistin use to 9% [105,106]. However, the enthusiasm generated by these studies is tempered by the observations according to which resistance to CAZ/AVI often occurs in CRE isolates, involving either the production of MBLs [104], mutations in different KPC enzymes [107,108], decreased outer membrane permeability, or enhanced drug efflux [109]. Therefore, all this information suggests the need to use the CAZ/AVI combination with caution in managing CRE infections.

IMI/REL gained FDA approval in July 2019, whereas MER/VAB was approved for use in the USA in 2017 [266]. Interestingly, it has been observed that adding REL, an AVI-like BLI, to IMI can increase the susceptibility of CRE isolates from 8% to 88% to IMI [120]. In general, IMI/REL has good activity on class A and class C β-lactamases. However, information on CRE susceptibility levels to IMI/REL remains conflicting. For example, a study conducted by Livermore et al. demonstrated a modest efficacy of IMI/REL on *K. pneumoniae* VIM, IMP, and NDM-producing strains [123]; in contrast, two other independent studies reported a 100% susceptibility on KPC-2 and KPC-3-producing *K. pneumoniae* isolates [120,124]. IMI/REL also proved its potency against CAZ/AVI-resistant *K. pneumoniae* isolates that harbored OXA-48-type carbapenemase. This study highlighted that IMI/REL and CAZ/AVI have overlapping spectra of action. These therapies can be used in conjunction to improve the therapeutic management of CRE infections [120]. Similarly, MER/VAB combination’s efficacy and safety were tested in two clinical trials, TANGO I and TANGO II, showing results as good as for other compounds and antibacterial combinations used in managing CRE infections [128,129]. MER/VAB also has a pronounced activity on class A and class C β-lactamases. Recently, MER/ VAB has gained approval from the European Medicines Agency (EMA) to treat cUTI, acute pyelonephritis, cIAIs, HAP, VAP, and other infections caused by aerobic Gram-negative pathogens in adults with limited treatment options [130]. Although well analyzed in clinical trials, many more studies are needed on the long-term stability of MER/VAB and IMI/REL and on the potential for antimicrobial resistance, as reported in the case of abusive administration of CAZ/AVI [265].

Plazomicin (Achaogen) is a next-generation semisynthetic aminoglycoside acting against bacteria producing AMEs [161]. In contrast with other aminoglycosides, several studies report higher potency of plazomicin against KPC-producing Enterobacteriaceae [267]. For instance, one of these studies investigated collections of clinically relevant KPC-producers with resistance to aminoglycosides and observed inhibition using plazomicin, with an MIC90 value of ≤2 mg/L [169]. Despite these promising results, MBL-producers are resistant to this antibiotic due to the methyltransferase enzymes commonly found, especially in NDM-producers [169]. Although aminoglycosides are not generally used as monotherapy, the broad spectrum of activity and low renal toxicity of plazomicin make it a choice for a targeted monotherapy against extensively-drug resistant Enterobacteriaceae causing UTI [173].

Another recently approved antibiotic in the fight against CRE is cefiderocol, also known as S-649266 [143]. According to the latest data, cefiderocol has been noted as one of the most potent antimicrobial compounds in the fight against fermenting and non-fermenting Gram-negative pathogens, including CRE [144]. Cefiderocol differs from all the agents mentioned above in that it provides activity against all four classes of β-lactamases [265]. In the ARGONAUT-I study, cefiderocol demonstrated in vitro superior antibacterial activity to the comparator drugs (e.g., cefepime, aztreonam, ceftazidime, CAZ/AVI, and CEF/TAZ) in combating CRE infections [268]. Additionally, in this study, it was observed that the type of the carbapenemase expressed by CRE influenced the activity of cefiderocol with MICs of 1 mg/L for strains expressing OXA-48-like genes, 2 mg/L for KPC-3, and 8 mg/L for strains producing NDM, KPC-2, and ESBLs [268]. Moreover, Karlowsky et al. showed that at a concentration of ≤4 mg/L, cefiderocol possesses in vitro activity against over 99% of *Enterobacteriaceae* strains resistant to CAZ/AVI ceftolozane/tazobactam, cefepime, ciprofloxacin, and colistin [147]. Despite the promising in vitro results of cefiderocol, the expected role in clinical practice is unclear due to the findings of higher all-cause mortality compared to the best available therapy for managing life-threatening infections caused by CRE [150]. In contrast with the presented agents, cefiderocol remains the only agent with activity against class B enzymes and may have a role in treating these infections. However, additional active agents are currently in the pipeline, including aztreonam combinations such as AZT/AVI [265].

AZT/AVI is a particular compound combining two FDA-approved agents: AZT and CAZ/AVI, that exerts activity on CPE and MBLs [153]. AZT is mainly resistant to the action of MBLs; however, aztreonam can be readily hydrolyzed by Ambler class A or class C β-lactamases [152]. Notably, avibactam can inhibit these secondary β-lactamases, which is why the AZT-AVI combination may be a valuable therapeutic strategy against MβL-producing organisms with secondary β-lactamases [153]. Case reports describe successful combinations of aztreonam, including CAZ-AVI, in the management of CRE infections. In a collection of 70 aztreonam non-susceptible CPE isolates, the addition of AVI restored their sensitivity to AZT [152]. Overall, for the entire collection of 70 isolates, the combined MIC90s were >64 mg/L for aztreonam and 2 mg/L for AZT/AVI combinations, this dose of AZT/AVI formulation blocking bacterial growth for approximately 98.6% of CRE strains [152]. Moreover, another study aiming to analyze the in vitro activity of CAZ-AVI and AZT/AVI, respectively, on CP-CRE and non-CP-CRE strains reported that CAZ/AVI was more potent against CRE isolates than AZT/AVI [155]. However, when compared to CAZ/AVI, AZT/AVI was less potent against non-CP-CRE isolates. The inoculum effect was much more prominent with AZT/AVI than in CAZ/AVI (47% and 18%, respectively), especially in *K. pneumoniae* isolates. Therefore, the use of the AZT/AVI combination should be performed with caution in the clinical context since the presence of a substantial inoculum effect may contribute to the clinical failure of high-inoculum infections treated with AZT/AVI [155].

Eravacycline, omadacycline and delafloxacin are new synthetic agents recently approved by the FDA and commercially available for treating infections caused by MDR microorganisms, acute bacterial skin and skin structure infections and community-acquired bacterial pneumonia. These agents demonstrated greater in vitro and in vivo potency and a clinical efficacy high as that of the comparator drugs (e.g., vancomycin, moxifloxacin, ceftriaxone, linezolid, levofloxacin) [183,188,189,203].

## 7. Conclusions

BLAs remain at present one of the most efficient antibiotic classes against MDR pathogens. Third generation penicillins (aminopenicillins, carboxypenicillins), the fifth generation of cephalosporins, and newly added cefiderococol are the most effective BLAs against MDR Gram-negative bacteria. The discovery of novel antibiotics, counteracting antimicrobial resistance through BLIs, is a promising strategy that could amplify these antibiotics’ action against CPE. Clinical trials have also revealed that CAZ-AVI, IMI-REL, and MEM-VAB are some of the most potent formulations in the fight against MDR-CPE. However, further studies in establishing new potent inhibitor formulations and their validation in clinical trials are required.

Future perspectives such as non-antibiotic drugs administered alone or in conjunction with classical antibiotics can be efficient in the fight against antimicrobial resistance. Other promising strategies exploited to reverse antibiotic resistance comes from liquid ion-based antimicrobial agents (ILs), machine learning approaches, and synthetic biology. More research is needed to find compounds with optimal biodegradability and efficient approaches to redesign antibiotic molecules in order to overcome antimicrobial resistance.

In the case of patients harboring CRE infections, physicians are undoubtedly left in a challenging situation: either they continue treating patients with older drugs with well-known drawbacks, or they adopt the new antibiotics, despite their higher costs and insufficient evidence of effect against CRE. A fine balance between these two treatment options is most likely to be the current best strategy. Despite that, more significant efforts must be devoted to designing and performing randomized clinical trials for CRE treatment.

## Figures and Tables

**Table 1:** Current promising antibiotics in treating carbapenem-resistant *Enterobacterales* (CRE) infections ^1^.

Drug	Mechanism of Action	Commercially Available	Indications	Limitations
Ceftazidime/avibactam	Cell wall synthesis inhibitor	Yes; FDA-approved in 2015 as Allergan	cUTI, cIAI, BSI, pneumonia	Occurrence of resistance
Ceftolozane/tazobactam	Inhibition of PBPs	Yes; FDA-approved in 2014 (Zerbaxa)	cUTI, cIAI	Occurrence of resistance
Meropenem/varbobactam	Cell wall synthesis inhibitor	Yes; FDA-approved in 2017 as Vabomere	cUTI, cIAI, BSI, pneumonia	Ocurrence of resistance
Ceftaroline/avibactam	Inhibition of PBPs	Under clinical investigation	cUTI	Occurrence of resistance due to mutations in KPC-producing *Enterobacteriaceae*
Cefepime/Zidebactam (WCK 5222)	Direct inhibition of β-lactamases or PBP2 inhibition	Under clinical development	cUTI, cIAI, SI, pneumonia	Ocurrence of resistance
Imipenem/cilastatin-relebactam	Renal dehydro-peptidase inhibitor/β-lactamase inhibitor	Yes; FDA-approved in 2019 as Recarbrio	cUTI, cIAI, pneumonia	Severe hypersensitivity reactions
Aztreonam/avibactam	Cell wall synthesis inhibitor	Under clinical development	cIAI	Likelihood of resistance among MBL- and AmpC-co-producing *K. pneumoniae*
Meropenem/nacubactam	Cell wall synthesis inhibitor	Under clinical investigation	cIAI	Occurrence of resistance; alterations of renal function
Plazomicin	Protein synthesis inhibitor	Yes, FDA-approved in 2018 as ZEMDRI	cUTI, BSI, pneumonia	Ineffective against MBL-producers
Eravacycline	Protein synthesis inhibitor	Yes, FDA-approved in 2018 as XERAVA	cIAI, pneumonia	Not indicated for the treatment of cUTI
Cefiderocol	Cell wall synthesis inhibitor	Yes, FDA-approved in 2019 as Fetroja	cUTI	Under investigation in clinical trials
Omadacycline	Protein synthesis inhibitor	Yes, FDA-approved in 2018 as NUZYRA	cUTI, pneumonia, acute SI	Limited action against ESBL-producing *K. pneumoniae*
Delafloxacin	Protein synthesis inhibitor (topoisomerase IV and DNA gyrase)	Yes, FDA-approved in 2017 as Baxdela (USA); Quofenix (EU).	Acute SI, pneumonia	Peripheral neuropathy and central nervous system effects

^1^ cUTI, complicated urinary tract infections; cIAI, complicated intra-abdominal infections; BSI, bloodstream infections; SI, skin infections; PBP, penicillin-binding protein; KPC, *Klebsiella pneumoniae* carbapenemase.
